# The Impact of Plasma Protein Binding Characteristics and Unbound Concentration of Voriconazole on Its Adverse Drug Reactions

**DOI:** 10.3389/fphar.2020.00505

**Published:** 2020-04-24

**Authors:** Zi-Qing-Yun Yuan, Chun Qiao, Zhi-Cheng Yang, Lei Yu, Lu-Ning Sun, Yi Qian, Xue-Hui Zhang, Ling Meng, Xiao-Yan Zhang, Yong-Qing Wang

**Affiliations:** ^1^Research Division of Clinical Pharmacology, First Affiliated Hospital of Nanjing Medical University, Nanjing, China; ^2^Hematology Department, First Affiliated Hospital of Nanjing Medical University, Nanjing, China; ^3^Department of Pharmacy, the Affiliated Jiangsu Shengze Hospital of Nanjing Medical University, Suzhou, China

**Keywords:** voriconazole, protein binding rate, unbound drug concentration, albumin, α1-acid glycoprotein

## Abstract

This study investigated voriconazole (VRC) unbound plasma concentration and its relationship with adverse drug reactions (ADRs) in patients with malignant hematologic disease. Plasma samples were collected from patients or spiked *in vitro*. A time-saving rapid equilibrium dialysis assay was used for the separation of unbound and bound VRC, following a high performance liquid chromatography-tandem mass spectrometry (HPLC-MS/MS) analysis method for drug concentration detection. Liver function and treatment details were collected from the electronic medical records of patients. Protein concentration was determined according to instructions. VRC plasma protein binding rate (PPB) in patient is significantly higher [69.5 ± 6.2%] than that in in-vitro samples, influenced by total drug concentration (C_t_), plasma protein concentration, and protein type. The α1-acid glycogen (AAG) has the highest affinity with VRC. Relationship between total PPB of VRC with PPB of individual protein is not a simple addition, but a compressive combination. Unbound drug concentration (C_u_) of VRC shows significant relationships with C_t_, protein concentration, AST level, metabolism type of CYP2C19 and co-administration of high PPB medicines. Unbound plasma concentration of VRC shows a more sensitive relationship with ADRs than C_t_.

## Introduction

Voriconazole (VRC), a second generation triazole anti-fungal agent with a spectrum against invasive aspergillosis and *Candida albicans* infections, has a broader spectrum of activity than traditional triazole agents (fluconazole and itraconazole), and is metabolized mainly by cytochrome P450 2C19 (CYP2C19) (Adwan, 2017). As the first-line treatment for invasive aspergillosis, VRC exhibits extreme inter-individual and intra-individual variation both in its efficacy and toxicity.

As is known to all, almost all drugs bind with plasma protein partially, while only unbound drug exerts pharmacological activity (Roberts et al., 2013). Plasma protein binding rate (PPB) of VRC was reported to be about 58% in premarketing studies previously (Roffey et al., 2003). Recently, unbound drug concentration was noticed by clinicians and pharmacists. The plasma binding characteristics of VRC was firstly investigated by Florent et al. (2014) in 2013. They found a high correlation between the total concentration (C_t_) and unbound concentration (C_u_). Unbound fraction (f_u_) was 32.3 ± 5.5% in blank plasma spiked with VRC *in vitro* and about 22.95% in plasma of patient treated with VRC, respectively. PPB of VRC has been investigated more in detail by Vanstraelen et al. (2014a) wherein the median PPB was about 47.6% in in-vitro samples and 49.6% in ICU samples, and it did not depend on the total drug concentration. VRC mainly binded to albumin (25.5 ± 5.1%), and to a lesser extent to α1-acid glycoprotein (AAG; 4.8 ± 1.2%). They also investigated the impact of hypoalbuminemia (< 35 g/liter) on VRC pharmacokinetics in adult ICU patients. A positive relationship occurred between voriconazole plasma protein binding rate and plasma albumin concentrations (*p* < 0.001), indicating higher unbound voriconazole concentrations with decreasing albumin concentrations (Vanstraelen et al., 2014b).

Although the plasma protein binding characteristics of VRC have been investigated by Vanstraelen et al. and Florent et al., results of these two studies are not consistent. C_u_ has rarely been investigated as a factor leading to variations in efficacy or toxicity of VRC, but factors that would influence the C_u_ or protein-binding characteristics of VRC is still not clear. The objectives of this article were to explore the protein binding characteristics of VRC and its influencing factors, to explore the factors influencing C_u_ (including C_t_, CYP2C19 metabolism type, co-administered medicine and liver function), and to explore the association of ADRs with unbound VRC plasma concentration.

## Methods

### Patient Blood Sample Collection

A non-controlled study (study A), approved by the Ethical Committee of Jiangsu Province Hospital (approved number: 2015-SR-206), was undertaken actively, carefully, and scientifically on the basis of Helsinki Declaration. Subjects were selected from patients treated in the hematology department of the First Affiliated Hospital of Nanjing Medical University/Jiangsu Province Hospital that with malignant blood diseases required VRC for treatment or prevention of invasive fungal infection. A total of 193 patients with signed informed consent participated in and completed this study. Detailed demographic information is shown in **Table 1**. All the patients were administered according to the dispensatory of Vfend^®^ that a loading dose of 6 mg/kg every 12 hours for two doses, followed by a maintenance dose of 4 mg/kg every 12 hours. Blood samples were collected from all 193 patients. A 5-ml Na-Heparin tube (Becton Dickinson, U.S.) was used for blood sample collection before (trough plasma concentration, C_min_) and 2 hours after (peak plasma concentration, C_max_) VRC administration (the endpoint of intravenous drip). After centrifugation (3500 rpm, 5 min), plasma was obtained from blood sample and was stored at −80°C until analysis.

**Table 1:** Demographic information of 193 patients.

Gender	Age (year)	Height (cm)	Weight (kg)	BMI (kg∙m^−2^)
Male (n = 105)	53.8 ± 17.3	169.5 ± 7.3	67.7 ± 10.7	23.5 ± 3.0
Female (n = 88)	51.3 ± 17.6	159.9 ± 5.0	58.5 ± 9.1	22.4 ± 3.4
Total (N = 193)	52.7 ± 17.4	165.6 ± 8.0	63.5 ± 11.0	23.0 ± 3.2

Causality, seriousness, and severity of ADRs were assessed by clinicians. Causality was assessed according to WHO-UMC criteria (World Health Organization, Uppsala Monitoring Centre). The specific type of ADRs was determined by referring to the specification of Vfend^®^ and previous literature (Levine and Chandrasekar, 2016). Seriousness and severity of ADRs were assessed based on appropriate clinical evaluation criteria for corresponding symptoms (e.g. scale).

### Metabolic Type Grouping

The DNAs of all patients were extracted by using Relax Gene Blood DNA System kit (Tiangen, China) and stored at −80℃ until the genotyping was performed. Sequencing of polymorphisms (*rs4244285, rs4986893, rs12248560* of CYP2C19) was conducted by Sangon Biotech (Shanghai, China). Only 124 patients were sequenced successfully, and were grouped into three different metabolic types (*1/*1, homozygous extensive metabolizer; *1/*2 or *1/*3, heterozygous extensive metabolizer; *2/*2, *2/*3 or *3/*3, poor metabolizer) for further analysis.

### Chemicals and Solutions

Voriconazole, provided by Tokyo Chemical Industry (Japan), was used to prepare a stock solution of 1.00 g/L in methanol (LC-MS grade from Merck, Germany). This stock solution was further diluted in acetonitrile or deionized pure water to a series of appropriate concentrations (working solution A and B, respectively). Working solution A was used for HPLC-MS/MS analysis, and B was for *in vitro* sample preparation. Fluconazole (internal standard, IS), provided by Sigma-Aldrich (USA), was used to prepare the IS working solution with acetonitrile (4.00 mg/L). Standard protein solutions were prepared in phosphate-buffered saline (PBS, containing 30 mmol/L sodium dihydrogen phosphate, 70 mmol/L disodium hydrogen phosphate, and 150 mmol/L sodium chloride), with Behring^®^ (CSL Behring GmbH, Marburg, Germany), α1-Acid Glycoprotein from human plasma (Sigma, Belgium) and Human Immunoglobulin (PH4) for Intravenous Injection (Chengdu Rongsheng pharmaceutical Co. Ltd). All solutions were freshly prepared at the moment of experiments.

### In Vitro Sample Preparation

Five *in vitro* studies (study B–F) were conducted for further investigation. Pooled blank lithium heparin plasma was collected from healthy volunteers. In study B, blank human plasma was spiked with VRC, resulting in four final concentrations (400 ng/ml, 1,500 ng/ml, 16,000 ng/ml, or 25,000 ng/ml). In study C, two concentrations of albumin (ALB) were spiked with two concentrations of VRC, resulting in four different ALB solution containing VRC (400 or 25,000 ng/ml of VRC in 30 or 50 g/L of ALB, respectively). Study D was similar to study C with two different concentrations of α1-acid glycoprotein (AAG) spiked with VRC resulting in four types of samples (400 or 25,000 ng/ml in 0.01 or 1 g/L of AAG, respectively). In study E, globulin (GLB) solutions were spiked with VRC resulting in four different final concentrations (400 or 25,000 ng/ml, in 10 or 30 g/L of GLB respectively). Study F included samples in two mixtures made up of different human plasma protein concentration (40.01 g/L [30 g/L ALB+10 g/L GLB+0.01 g/L AAG] or 81 g/L [50 g/L ALB+30 g/L GLB+1 g/L AAG]) with two drug concentrations (400 or 25,000 ng/ml), resulting in four final mixtures (400 or 25,000 ng/ml in 40.01 or 81 g/L of protein mixtures).

### Unbound VRC Separation

Unbound and bound VRC of all plasma samples from patients and *in vitro* samples were separated using rapid equilibrium dialysis (RED, Thermo Fisher Scientific, USA)(Qian et al., 2019). In brief, unbound VRC was separated from plasma in a 96-well plate with a semipermeable membrane through which only unbound drug can permeate. Equilibrium was reached after 2 hours at room temperature in a mixer at 800 rpm according to the specification.

### Analytical Methods for the Buffer and Plasma Concentration of Voriconazole

After reaching equilibrium, VRC concentrations in both the plasma and the buffer compartment were determined using a validated HPLC-MS/MS method, with a LLOQ of 50.00 ng/ml, between-run precision, expressed as coefficient of variation, below 15%, and accuracy, expressed as a percentage of the theoretically added concentration, of between 88% and 107%. Linearity from 50.00 to 30,000 ng/ml was demonstrated, and a second order calibration model was employed which provided coefficients of determination (r^2^) better than 0.997.

Plasma samples and the buffer compartment were prepared using a protein precipitation method. In short, 200 μl acetonitrile (containing internal standard, 4 mg/L) were mixed with 50 μl plasma sample (or the *in vitro* sample). 100 μl of clear upper layer was diluted with 900 μl deionize water after centrifugation (16,000 rpm, 15 min, 4℃). After vortex, the mixture was injected into the HPLC system for separation and then the separated components are detected by MS and quantification was made through calibration curve.

Concentrations were quantified by using a C18 reversed-phase chromatogram column Hypersil^®^ GOLD Dim (Thermo Fisher Scientific, 2.1 mm × 50 mm, 1.9 μm) with a tandem mass spectrometer Finnigan TM TSQ Quantum Discovery MAXTM (Thermo Fisher Scientific, USA), operated in positive-ion electrospray mode. The mobile phase consisted of 0.2% (v/v) formic acid and methanol (45:55, v/v). Column temperature was 35°C and run-time was set at 6.0 min. Compounds were detected *via* selected reaction monitoring (SRM) mode (voriconazole m/z 350.2 →281.0, fluconazole m/z 301.1→238.1). Electrospray ionization (ESI) interface parameters were as follows: spray voltage 3500 V, sheath gas, and auxiliary gas (N_2_) 35 and 15 arbitrary units respectively, capillary heater temperature 350℃, collision energy (voriconazole 20 V, fluconazole 16 V), collision gas 1.5 mTorr. The volume of injection is 3 μl. The method was validated within the linear range 50.00 to 30,000 ng/ml.

### Calculation of PPB of Voriconazole

VRC plasma protein bounding rate (PPB) was calculated according to the following formula:

$$\text{PPB}\%=\frac{C_{plasma}-C_{buffer}}{C_{plasma}}\times 100\%$$

C_buffer_ and C_plasma_ represent the concentration of voriconazole at equilibrium in the buffer compartment and plasma compartment, respectively.

### Clinical Data Collection

Treatment details (adverse effects and concomitant medication) and liver function tests (serum concentrations of alanine transaminase [ALT], aspartate transaminase [AST]) were collected from the electronic medical records of patients.

### Concentration Analysis for Total Protein, ALB, and AAG

Plasma total protein (TP), ALB, and AAG concentrations of all plasma samples from patients were determined by BCA protein quantification kit (Beyotime, China), bromocresol green albumin assay kit (Nanjing Jiancheng Bioenjineering Institute, China), and colorimetric sandwich ELISA kit (ProteintechGroup, USA) according to specifications, respectively.

### Statistical Analysis

Statistical analysis was performed by using SPSS 22.0 for Windows (SPSS Inc. 2011, Chicago, Illinois). One-way analysis of variance (ANOVA) and t-test were used to compare the difference in mean; Pearson correlation analysis was used to test the correlation between the factors (plasma protein concentration and VRC plasma concentration) and PPB of VRC in patient plasma samples. Data were described with mean ± standard deviations (SD). All the statistical tests were two-sided and considered as significant for a *p* value less than 0.05.

## Results

**Figure 1:**
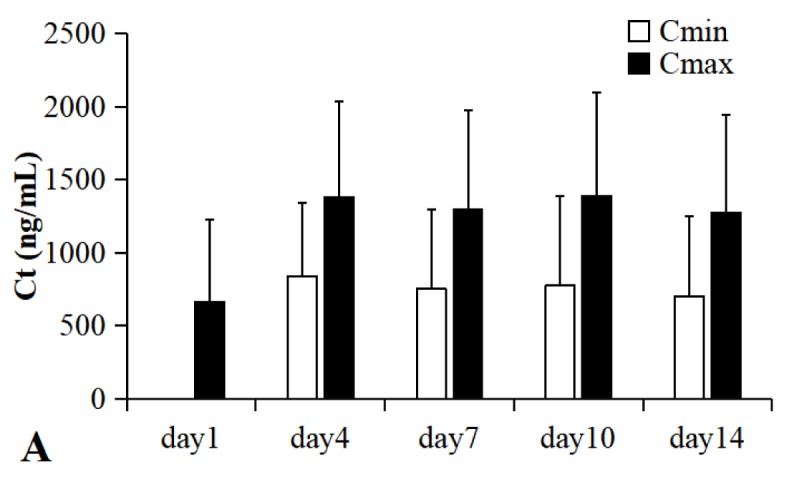
Total drug plasma concentration of VRC from 193 patients with malignant hematologic disease in day 1, 4, 7, 10, 14 (n=240, 230, 214,142, 106 respectively). C_t_, total drug concentration; C_min_, trough plasma concentration; C_max_, peak plasma concentration. All patients were administered by intravenous drip (2 hours), 4 mg/kg (twice a day) after a loading dose of 6 mg/kg (every 12 h) in day 1. Both the C_min_ and the C_max_ of VRC reached the steady state from day 4.

Demographic information was shown in **Table 1**. Total drug concentration (C_t_) of VRC in 932 plasma samples from 193 patients collected on day 1, 4, 7, 10, 14 (n=240, 230, 214,142, 106 respectively) was shown in **Figure 1**. Both C_min_ and C_max_ of VRC reached the steady state on day 4.

Protein binding rate of VRC in different sample type is significantly different, except that between study B and F, study C and D, and study C and E (shown in **Table 2**). Protein binding rate in plasma of patient was the highest (69.5 ± 6.2%), while that in GLB solutions spiked with VRC was the lowest (37.8 ± 4.0%).

**Table 2:** Protein binding rates among different sample types.

Samples		n	PPB% (Mean ± SD)	*p*
***In vivo* samples**		79	69.5 ± 6.2	vs. healthy human plasma, *p* < 0.01;
				MIX, *p* < 0.01;
				ALB, *p* < 0.01;
				ACG, *p* < 0.01;
				GLB, *p* < 0.01;
***In vitro* samples**	VRC spiked with healthy human blank plasma	80	58.0 ± 4.7	vs. MIX, *p* = 0.066
				ALB, *p* < 0.01;
				ACG, *p* < 0.01;
				GLB, *p* < 0.01;
	VRC spiked with protein MIX (ALB+AAG+GLB)	24	56.1 ± 4.6	vs. ALB, *p* < 0.01;
				ACG, *p* < 0.01;
				GLB, *p* < 0.01;
	VRC spiked with human ALB	42	42.9 ± 6.3	vs. ACG, *p* = 0.020;
				GLB, *p* < 0.01;
	VRC spiked with human AAG	36	46.5 ± 7.6	vs. GLB, *p* < 0.01
	VRC spiked with human GLB	24	37.8 ± 4.1	

VRC, voriconazole; ALB, albumin; AAG, α1-acid glycoprotein; GLB, globulin; PPB, plasma protein binding rate.

### Plasma Protein Binding Characteristics of VRC in Patient Plasma

**Figure 2:**
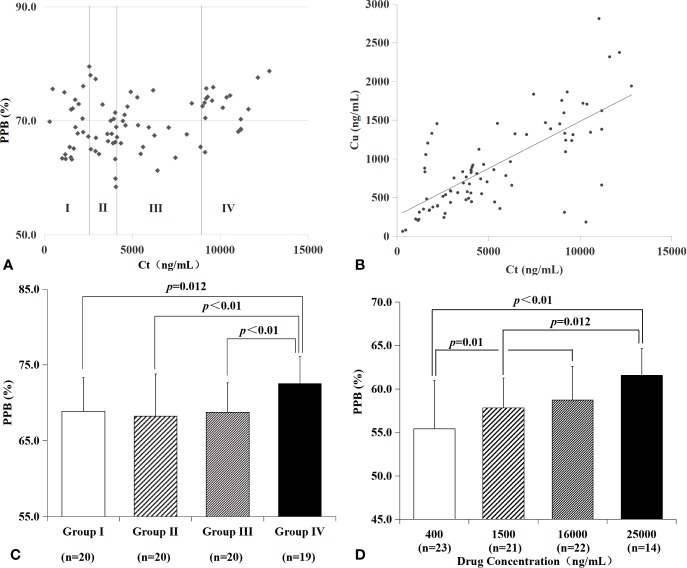
PPB and C_u_ characteristics of VRC in plasma from patients and blank plasma spiked with drugs in different C_t_. There was a trend of positive relationship between C_t_ and PPB (**A**, R=0.291), C_t_ and C_u_ (**B**, R=0.724) in plasma from patients. Patients with a higher C_t_ in group IV had a significantly higher PPB (**C**, group I vs. group IV, p=0.012; group II vs. group IV, *p* < 0.01; group III vs. group IV, p<0.01). Blank plasmas spiked with VRC *in vitro* were divided into four groups according to drug concentration **(D)**. There was a significant lower PPB of 400 ng/ml VRC than 16,000 ng/ml VRC (p=0.01), or 25,000 ng/ml VRC (p<0.01); 1,500 ng/ml VRC had a significantly lower PPB than 25,000 ng/ml (p=0.012).

There was a trend of positive relationship between C_t_ and PPB (shown in **Figure 2A**, p<0.01, R=0.291), C_t_ and C_u_ (shown in **Figure 2B**, p<0.01, R=0.724) according to Pearson correlation analysis. The PPBs with C_t_ in 79 plasma samples from 41 patients, shown in **Figure 2A** (scatter plot), were divided into four groups according to quartiles of C_t_ (group I, 0–2559 ng/ml, n=20; group II, 2,559-4,114 ng/ml, n=20; group III, 4,114–8,944 ng/ml, n=20; group IV, more than 8,944 ng/ml, n=19). Patients with a higher C_t_ (more than 8,944 ng/ml) in group IV had a significantly higher PPB (group I vs. group IV, p=0.012; group II vs. group IV, p<0.01; group III vs. group IV, p<0.01, shown in **Figure 2C**).

### Protein Binding Characteristics of VRC From In Vitro Studies

All of the blank plasma spiked with VRC *in vitro* (n=80) were divided into four groups according to total drug concentration (400, 1,500, 16,000, and 25,000 ng/ml, shown in **Table 3** and **Figure 2D**). Protein binding rates were significantly different between 400 and 16,000 ng/ml (p=0.010), 400 and 25,000 ng/ml (*p* < 0.01), 1,500 and 25,000 ng/ml (p=0.012), respectively.

**Table 3:** PPBs among different *in vitro* samples grouped by C_t_.

Sample type	C_t_	C_p_	n	PPB%	*p*		C_u_	*p*	
	(ng/ml)	(g/L)		(Mean ± SD)			(ng/ml)		
**VRC spiked with human blank plasma**	Total	–	80	58.0 ± 4.7	–		–	–	
	25,000		14	61.6 ± 3.1	25 vs. 16	0.012	6777 ± 874	–	
	16,000		22	58.7 ± 3.9	16 vs. 0.4	0.010	4825 ± 888		
	1,500		21	57.8 ± 3.4			481.5 ± 96.5		
	400		23	55.4 ± 5.6	0.4 vs. 25	<0.01	118.4 ± 19.8		
**VRC spiked with human ALB**	Total		42	42.9 ± 6.3	–		–	–	
	25,000	Total	24	43.7 ± 6.8	–		6391 ± 357	–	
		30	8	36.8 ± 6.0	30 vs. 40	0.001	6459 ± 410	>0.05	
		40	8	45.5 ± 4.1			6300 ± 208		
		50	8	48.7 ± 3.9	50 vs. 30	<0.01	6416 ± 437		
	400	Total	18	41.9 ± 5.4	–		110.8 ± 10.8	–	
		30	6	44.2 ± 6.6	>0.05		109.5 ± 5.3	>0.05	
		40	6	39.0 ± 4.6			113.6 ± 13.5		
		50	6	42.5 ± 4.3			109.3 ± 13.1		
**VRC spiked with human AAG**	Total		36	46.5 ± 7.6	–		–	–	
	25,000	Total	18	45.5 ± 8.8	–		6690 ± 729	–	
		0.01	6	36.9 ± 7.7	0.01 vs. 0.1	0.015	7305 ± 435	0.01 vs. 1	<0.01
		0.1	6	45.6 ± 4.0	0.1 vs. 1	0.018	6946 ± 338	0.1 vs. 1	<0.01
		1	6	54.0 ± 3.7	1 vs. 0.01	<0.01	5817 ± 240		
	400	Total	18	47.5 ± 6.3	–		105.2 ± 6.8	–	
		0.01	6	40.9 ± 3.3	0.01 vs. 0.1	0.003	108.9 ± 4.2	>0.05	
		0.1	6	50.0 ± 2.8			100.1 ± 2.5		
		1	6	51.5 ± 6.3	1 vs. 0.01	0.001	106.5 ± 9.0		
**VRC spiked with human GLB**	Total	–	24	37.8 ± 4.1	–		–	–	
	25,000	Total	12	36.2 ± 3.4	–		6859 ± 493		
		10	6	35.2 ± 4.3	>0.05		7251 ± 338	0.001	
		30	6	37.2 ± 2.0			6467 ± 224		
	1,500	Total	12	39.4 ± 4.3	–		419.0 ± 22.1	–	
		10	6	39.5 ± 2.7	>0.05		418.5 ± 20.9	>0.05	
		30	6	39.4 ± 5.8			419.6 ± 25.2		
**VRC spiked with ALB+AAG+GLB**	Total	–	24	56.1 ± 4.6	–		–	–	
	25,000	Total	12	59.0 ± 2.9	–		6042 ± 82	–	
		40.01	6	55.7 ± 0.9	<0.01		6009 ± 90	>0.05	
		81	6	59.6 ± 1.6			6097 ± 72		
	400	Total	12	53.2 ± 4.2	–		121.9 ± 5.6	–	
		40.01	6	47.8 ± 1.4	<0.01		127.5 ± 4.2	<;0.01	
		81	6	55.4 ± 1.5			117.0 ± 3.5		

VRC, voriconazole; ALB, albumin; AAG, α1-acid glycoprotein; GLB, globulin; C_p_, protein concentration; C_t_, total drug concentration

PPB, plasma protein binding rate.

**Figure 3:**
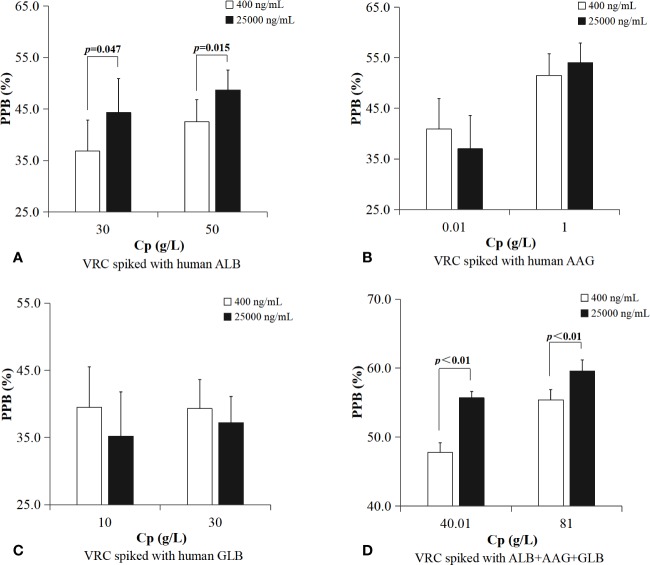
PPBs of plasma protein solutions spiked with VRC *in vitro* were grouped according to total drug concentration. ALB binding rate of samples with a higher concentration of VRC (C_t_=25,000 ng/ml) was significantly higher in different protein groups (**A**, 30 g/L, p=0.047; 50 g/L, p=0.015). Plasma protein mixtures spiked with a higher concentration of VRC (C_t_=25,000 ng/ml) had a significantly higher PPB than the lower concentration group (C_t_=400 ng/ml) in both two different protein groups (**D**, 40.01 g/L, p<0.01; 81 g/L, p<0.01). There was no difference in AAG or GLB binding rate between different drug concentration groups **(B, C)**.

PPBs of plasma protein solutions spiked with VRC *in vitro* (shown in **Table 3** and **Figure 3**) were grouped according to total drug concentration (C_t_). ALB binding rate of samples with a higher concentration of VRC (C_t_=25,000 ng/ml) was significantly higher in different protein groups (30 g/L, p=0.047; 50 g/L, p=0.015). There was no similar result in AAG or GLB binding rate. Plasma protein mixtures spiked with a higher concentration of VRC (C_t_=25,000 ng/ml) had a significantly higher binding rate than the lower concentration group (C_t_=400 ng/ml) in both two different protein groups (40.01 g/L, p<0.01; 81 g/L, p<0.01).

**Figure 4:**
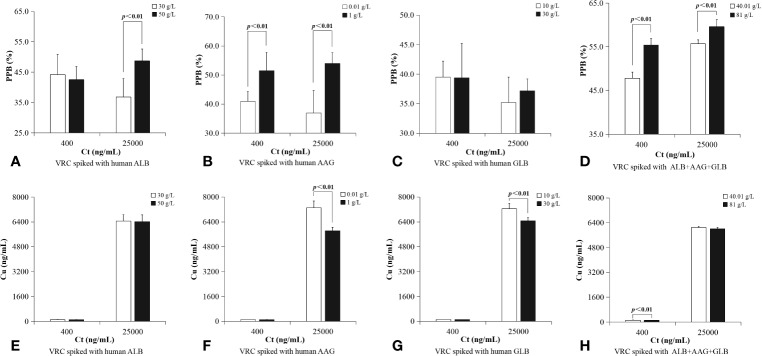
PPBs of plasma protein solutions spiked with VRC *in vitro* were grouped according to protein concentration. ALB binding rate of samples in higher C_t_ group (25,000 ng/ml) with higher protein concentration was significantly higher (**A**, 50 vs. 30 g/L, p < 0.01). AAG binding rate of samples with higher protein concentration had a significant higher PPB in both two C_t_ groups (**B**, C_t_=400 ng/ml, [0.01 vs. 1 g/L, p = 0.001]; C_t_=25,000 ng/ml, [0.01 vs. 1 g/L, p < 0.01]); GLB binding rate was without this characteristic in either lower C_t_ group or higher C_t_ group **(C)**. Plasma protein mixtures spiked with a higher concentration of protein (C_p_=81 g/L) had a significant higher PPB than the lower group (C_p_=40.01 g/L) in both two different C_t_ groups (**D**, C_t_=400 ng/ml, [40.01 vs. 81 g/L, p < 0.01]; C_t_=25,000 ng/ml, [40.01 vs. 81 g/L, p < 0.01]). There was no significant difference between lower ALB concentration and higher ALB concentration in either lower C_t_ group or higher C_t_ group **(E)**. In a higher C_t_ (25,000 ng/ml), C_u_ of a lower AAG concentration was significantly higher than samples with higher AAG concentration (**F**, 0.01 vs. 1 g/L, p < 0.01). In a higher C_t_ (25,000 ng/ml), C_u_ of *in vitro* samples with a lower GLB concentration (10 g/L) was significantly higher than samples with higher GLB concentration (**G**, 30 g/L, p < 0.01). In a lower C_t_ (400 ng/ml), C_u_ of a lower plasma protein concentration was significantly higher than sample with a higher plasma protein concentration (**H**, 40.01 vs. 81 g/L, p < 0.01).

PPBs of plasma protein solutions spiked with VRC *in vitro* were also grouped according to protein concentration (shown in **Table 4** and **Figure 4**). ALB binding rate of samples in higher C_t_ group (25,000 ng/ml) with higher protein concentration was significantly higher (50 vs. 30 g/L, p<0.01). AAG binding rate of samples with higher protein concentration had a significant higher PPB in both two C_t_ groups (C_t_=400 ng/ml, [0.01 vs. 1 g/L, p<0.01]; C_t_=25,000 ng/ml, [0.01 vs. 1 g/L, p<0.01]). GLB binding rate was without this characteristic in either lower C_t_ group or higher C_t_ group. Plasma protein mixtures spiked with a higher concentration of protein (C_p_=81 g/L) had a significant higher PPB than the lower group (C_p_=40.01 g/L) in both two different C_t_ groups (C_t_=400 ng/ml, [40.01 vs. 81 g/L, p<0.01]; C_t_=25,000 ng/ml, [40.01 vs. 81 g/L, p<0.01]).

**Table 4:** PPBs and C_u_ among different *in vitro* samples grouped by C_p_.

Sample Type	C_p_ (g/L)	C_t_ (ng/ml)	n	PPB (Mean ± SD%)	*p*
**VRC spiked with human ALB**	Total	–	42	42.9 ± 6.3	
	30	Total	14	40.0 ± 7.1	
		25,000	8	36.8 ± 6.0	0.047
		400	6	44.3 ± 6.6	
	40	Total	14	42.8 ± 5.3	
		25,000	8	45.5 ± 4.1	0.017
		400	6	39.1 ± 4.6	
	50	Total	14	46.0 ± 5.0	
		25,000	8	48.7 ± 3.9	0.015
		400	6	42.5 ± 4.3	
**VRC spiked with human AAG**	Total	–	36	46.5 ± 7.6	
	0.01	Total	12	38.9 ± 6.0	
		25,000	6	37.0 ± 7.7	>0.05
		400	6	40.9 ± 3.3	
	0.1	Total	12	47.8 ± 4.0	
		25,000	6	45.6 ± 4.0	
		400	6	50.0 ± 2.7	
	1	Total	12	52.8 ± 5.1	
		25,000	6	54.0 ± 3.7	
		400	6	51.5 ± 6.3	
**VRC spiked with human GLB**	Total	–	24	37.8 ± 4.1	
	10	Total	12	37.4 ± 4.1	>0.05
		25,000	6	35.2 ± 4.3	
		1,500	6	39.5 ± 2.7	
	30	Total	12	38.3 ± 4.3	
		25,000	6	37.2 ± 2.0	
		1,500	6	39.3 ± 5.8	
**VRC spiked with ALB+AAG+GLB**	Total	–	24	56.1 ± 4.6	
	40.01	Total	12	51.7 ± 4.3	
		25,000	6	55.7 ± 0.9	<0.01
		400	6	47.8 ± 1.4	
	81	Total	12	57.5 ± 2.6	
		25,000	6	59.6 ± 1.6	<0.01
		400	6	55.4 ± 1.5	

ALB, albumin; AAG, α1-acid glycoprotein; GLB, globulin; C_p_, protein concentration; C_t_, total drug concentration; C_u_, unbound drug concentration; PPB, plasma protein binding rate

**Figure 5:**
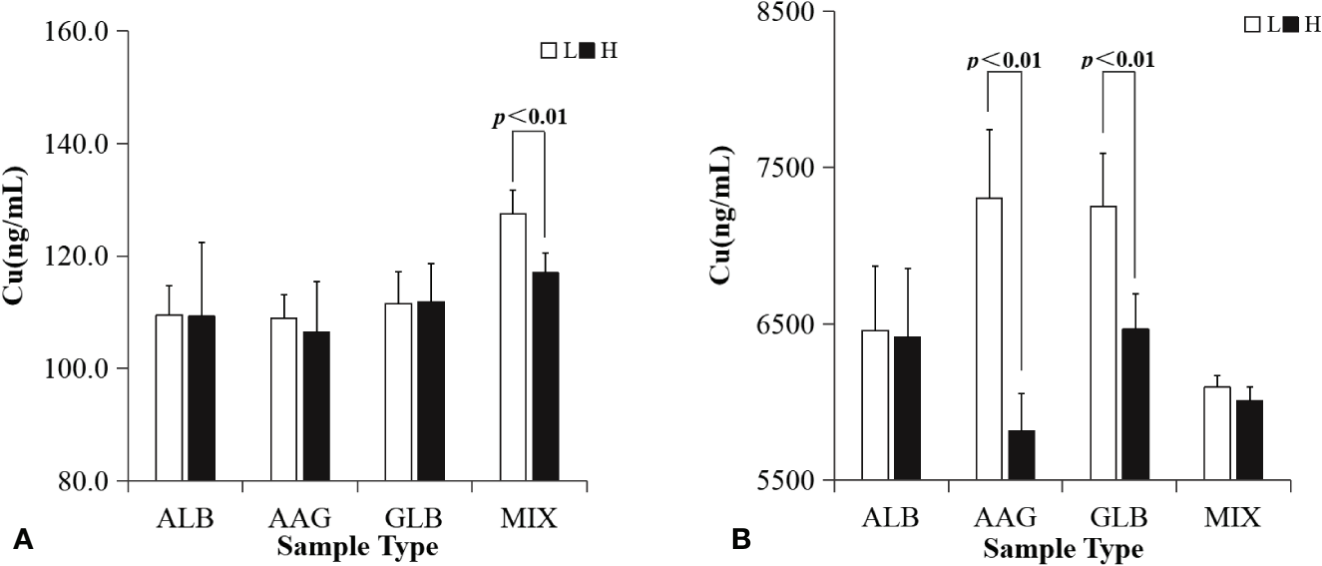
Unbound drug concentrations of plasma protein solutions spiked with VRC *in vitro* were grouped according to protein concentration. L or H represented *in vitro* samples in lower or higher C_p_, respectively. MIX represents the plasma protein mixtures *in vitro*. In a lower C_t_ (**A**, 400 ng/ml), C_u_ of *in vitro* samples with a lower plasma protein concentration was significantly higher than samples with a higher plasma protein concentration (40.01 vs. 81 g/L, p<0.01). In a higher C_t_ (**B**, 25,000 ng/ml), C_u_ of *in vitro* samples with a lower AAG concentration was significantly higher than samples with higher AAG concentration (0.01 vs. 1 g/L, p<0.01), and C_u_ of *in vitro* samples with a lower GLB concentration (10 g/L) was significantly higher than samples with a higher GLB concentration (30 g/L, p<0.01).

C_u_ characteristic of all *in vitro* samples were shown in **Table 4** and **Figure 5**. In a lower C_t_ (400 ng/ml), C_u_ of a lower plasma protein concentration was significantly higher than sample with a higher plasma protein concentration (40.01 vs. 81 g/L, p<0.01). In a higher C_t_ (25,000 ng/ml), C_u_ of a lower AAG concentration was significantly higher than samples with higher AAG concentration (0.01 vs. 1 g/L, p<0.01). In a higher C_t_ (25,000 ng/ml), C_u_ of *in vitro* samples with a lower GLB concentration (10 g/L) was significantly higher than samples with higher GLB concentration (30 g/L, p<0.01).

### Relationship Between Plasma Protein Concentration and C_t_, C_u_, and PPB in Plasma of Patients

PPB was correlated positively with AAG (p=0.013, R=0.314), and ALB showed a positive correlation with TP (p=0.031, R=0.461) according to Pearson relationship analysis. There was no other significant correlation between C_t_, C_u_, PPB, and plasma protein concentration, respectively.

### Plasma Concentration of VRC in Different Metabolic Types

**Figure 6:**
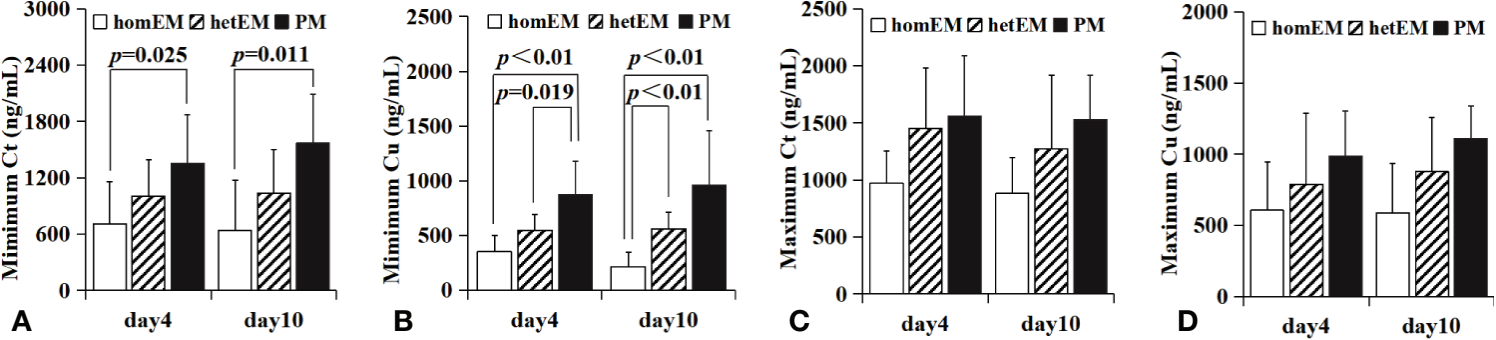
Relationship between metabolic type and drug concentration. homEM, homozygous extensive metabolizer; hetEM, heterozygous extensive metabolizer; PM, poor metabolizer. The total C_min_ of VRC in day 4 and day 10 in homEM [(day 4, n = 26, 712.7 ± 446.9 ng/ml), (day 10, n = 13, 641.2 ± 532.0 ng/ml)]was significantly lower than PM [(day 4, n = 11,1356.0 ± 519.6 ng/ml, p = 0.025), (day 10, n = 8, 1567.4 ± 523.0 ng/ml, p = 0.011), shown in **(A)**]. The unbound C_min_ of VRC in day 4 in PM [n = 6, 874.6 ± 304.6 ng/ml] was significantly higher than that in homEM [n = 6, 350.8 ± 152.5 ng/ml, p < 0.01] and hetEM [n = 6, 548.9 ± 144.0, p=0.019], shown in **(B)**. The unbound C_min_ of VRC in day 10 (shown in **B**) in homEM [n = 7, 215.2 ± 132.6 ng/ml] was significantly lower than that in hetEM [n = 6, 562.3 ± 147.7 ng/ml, p < 0.01] and PM [n = 6, 957.6 ± 502.8 ng/ml, p < 0.01]. There was no similar relationship between metabolic type and C_max_ (**C**, **D**).

Only 124 patients were sequenced successfully, and were grouped into three different metabolic types (*1/*1, homozygous extensive metabolizer; *1/*2 or *1/*3, heterozygous extensive metabolizer; *2/*2, *2/*3 or *3/*3, poor metabolizer).The total C_min_ of VRC on day 4 and day 10 (shown in **Figure 6A**) in homozygous extensive metabolizer (homEM) [(day 4, n=26, 712.7 ± 446.9 ng/ml), (day 10, n=13, 641.2 ± 532.0 ng/ml)] was significantly lower than those in poor metabolizer (PM) [(day 4, n=11,1356.0 ± 519.6 ng/ml, p=0.025), (day 10, n=8, 1567.4 ± 523.0 ng/ml, p=0.011)]. The unbound C_min_ of VRC on day 4 (shown in **Figure 6B**) in PM [n=6, 874.6 ± 304.6 ng/ml] was significantly higher than that in homEM [n=6, 350.8 ± 152.5 ng/ml, p<0.01] and hetEM [n=6, 548.9 ± 144.0, p=0.019]. The unbound C_min_ of VRC on day 10 (shown in **Figure 6B**) in homEM [n=7, 215.2 ± 132.6 ng/ml] was significantly lower than that in hetEM [n=6, 562.3 ± 147.7 ng/ml, p<0.01] and PM [n=6, 957.6 ± 502.8 ng/ml, p<0.01]. But there was no significant difference between different metabolic type in maximum drug concentration (shown in **Figures 6C, D**).

### Plasma Concentration Between Normal and Abnormal Hepatic Function

**Figure 7:**
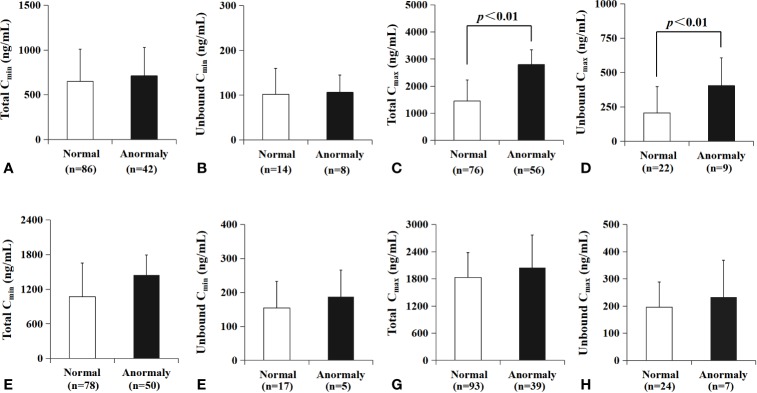
Relationship between AST or ALT level and drug concentration, respectively. There was no significant difference between patients with AST in normal value and those with abnormal AST (shown in **A, B**) in C_min_. The total C_max_ of VRC in patients with AST in normal value were significantly lower than those with abnormal AST value (p<0.01, **C**). The unbound C_max_ of VRC in patients with normal AST was lower than those with abnormal AST value (p<0.01, **D**). ALT values showed no significant influence on either C_t_ or C_u_ in minimum or maximum concentration **(E–H)**.

All plasma samples were divided into two groups according to hepatic function (the value of AST or ALT), respectively. There was no significant difference between patients with AST in normal value and those with abnormal AST (shown in **Figures 7A, B**) in the mean of C_min_.The total C_max_ in patients with AST in normal value [1453 ± 777 ng/ml, n=76] was significantly lower than those with abnormal AST [2795 ± 549 ng/ml, n=56, p < 0.01] (shown in **Figure 7C**). The unbound C_max_ of VRC in patients with normal AST [206.7 ± 191.8 ng/ml, n=22] was significantly lower than those with abnormal AST [405.4 ± 201.9 ng/ml, n=9, p < 0.01] (shown in **Figure 7D**). AST showed no significant influence on minimum concentration of VRC. ALT values showed no significant influence on either C_t_ or C_u_ in minimum or maximum concentration (shown in **Figures 7E–H**).

### Adverse Drug Reactions

**Figure 8:**
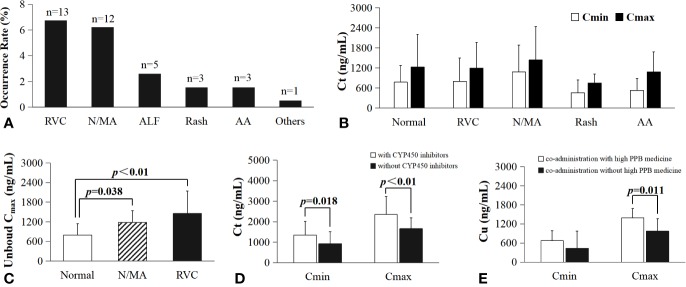
The impact of co-administration and the relationship of VRC drug concentration with ADRs. RVC, reversible visual changes; N/MA, nerval or mental abnormity; ALF, abnormal liver function; AA, abnormal audition; **(A)**, the occurrence rate and frequency of all ADRs; **(B)**, C_t_ (including C_max_ and C_min_) in patients with and without (normal group) ADRs; **(C)**, significant difference in patients unbound C_max_ with and without ADRs; the unbound C_max_ in patients without ADRs (792.8 ± 348.9 ng/ml, n=21) was significantly lower than those with nerval or mental abnormity (1187 ± 355 ng/ml, n=10, p=0.038), with reversible visual changes (1453 ± 693 ng/ml, n=13, p<0.01).; **(D)**, The C_t_ in patient co-administration with CYP450 inhibitor esomeprazole (C_min_=1355 ± 657 ng/ml, n=24; C_max_=2354 ± 867 ng/ml, n=33) was significantly higher than those without CYP450 inhibitor (C_min_=0.930 ± 0.581, n=318, p=0.018; C_max_=1.657 ± 0.530, n=429, p<0.01); **(E)**, significant difference of unbound C_max_ in plasma samples from patients co-administration with (1390 ± 541 ng/ml, n=28) or without high PPB medicine (971.1 ± 390.8 ng/ml, n=16, p=0.011).

The total rate of adverse drug reactions (ADRs) for VRC in this study was 16.6% (32 from 193 patients). Reversible visual changes (12 visual hallucinations and 1 blurred vision) occurred in 13 patients (6.7%). Neurological abnormity or mental abnormity (10 nightly excitement, 1 lack of alertness, and 1 unconsciousness) occurred in 12 patients (6.2%). Abnormal liver function occurred in 5 patients (2.6%). Rash occurred in 3 patients (1.6%). Abnormal audition occurred in 3 patients (1.6%), and cyclosporine plasma concentration increasing occurred in one patient (0.5%) when co-administered with cyclosporine (shown in **Figure 8A**).

There was no significant difference between C_t_ (including C_max_ and C_min_) in patients with and without ADRs (shown in **Figure 8B**), while the unbound C_max_ (shown in **Figure 8C**) in patients without ADRs (792.8 ± 348.9 ng/ml, n=21) was significantly lower than those with neurological abnormity or mental abnormity (1187 ± 355 ng/ml, n=10, p=0.038), or those with reversible visual changes (1453 ± 693 ng/ml, n=13, p<0.01).

### Drug-Drug Interaction

10 patients co-administered with CYP450 enzyme inhibitor esomeprazole. The C_t_ was significantly higher in patient used CYP450 inhibitor esomeprazole (C_min_=1355 ± 657 ng/ml, n=24; C_max_=2354 ± 867 ng/ml, n=33) in combination than those without CYP450 inhibitor (C_min_=930.4 ± 581.4 ng/ml, n=318, p=0.018; C_max_=1657 ± 530 ng/ml, n=429, p<0.01) shown in **Figure 8D**). Medicine with high protein binding rate, such as teicoplanin, pantoprazole, esomeprazole, lansoprazole, estazolam, ambroxol (PPB more than 90%), had been co-administered in most patients (except 28 patients). Unbound C_max_ in plasma samples from patients used high PPB medicine (1390 ± 541 ng/ml, n=28) in combination was significantly higher than those without high PPB medicine (971.1 ± 390.8 ng/ml, n=16, p=0.011, shown in **Figure 8E**).

## Discussions

Total drug concentration of VRC in 932 plasma samples from 193 patients were determined by a HPLC-MS/MS method. The C_max_ (1336 ± 676 ng/ml) in steady state of VRC reached therapeutic window (1,000–5,500 ng/ml) (Cabral-Galeano et al., 2015; Sano et al., 2018). However, there was still wide inter-individual variability in plasma concentration of VRC mostly associated with the diversity of metabolic types (CYP2C19) (Hamadeh et al., 2017).

Unbound concentration of 79 plasma samples were separated by RED, and showed a positive relationship with C_t_, which was consistent with the result from Florent et al. (Florent et al., 2014). From the result of our *in vitro* studies, C_u_ was also influenced by C_t_ and the protein concentration. Samples with a lower protein concentration or a higher C_t_ were associated with a significantly higher C_u_. It was influenced by some other factors, such as the CYP2C19 metabolic types, liver function of patients, and co-administration during the treatment.

CYP2C19 is the major metabolic enzyme involved in VRC metabolism (Yanagimoto et al., 2013). The minimum C_u_ in steady state of PMs were significantly higher than those of homEMs in our result. The similar relationship appeared in minimum C_t_, but the *p* value in C_u_ was lower which indicated that C_u_ may be more sensitive to the metabolic type of CYP2C19 than C_t_.

Apart from CYP2C19 metabolic types, the liver function also showed an impact on C_u_ because VRC is metabolized mainly in the liver. The levels of AST and ALT are reliable parameters for liver function (Sookoian and Pirola, 2015). Data analysis showed that the maximum C_t_ and C_u_ were both in a higher level when AST was in an abnormal situation. However, there was no similar result occurred in ALT values in our research. This might have been due to the co-administration with hepatoprotectants (glutathione, polyene phosphatidylcholine). ALT is mainly distributed in hepatocytes, so the variation of ALT may be influenced when used hepatoprotectants in combination. AST is mainly distributed in the cardiac, which may be influenced less by the liver function. These are different from the results reported from Vanstraelen et al. (2014a).

Subjects in this study were patients with malignant blood diseases. There were many concomitant medications during the treatment of malignant blood diseases. C_u_ of voriconazole may also be affected by these combinations. In our study, C_u_ was significantly higher in patients administered with medicine of high protein binding rate (more than 90%, such as teicoplanin (Yanagimoto et al., 2013), pantoprazole, lansoprazole, esomeprazole (Andersson and Weidolf, 2008), estazolam, and ambroxol).In addition, C_t_ was higher in patients administered with lansoprazole, which is a potent inhibitor of CYP2C19 that may have reduced the hepatic clearance of VRC (Lopez and Tayek, 2016). These results indicated that the concomitant medication may be a factor that influence the plasma concentration of VRC. The C_u_ in patients co-administered with or without lansoprazole should be analyzed if there was enough cases.

C_u_ was influenced by a lot of factors. In our study, the relationship between C_u_ and ADRs was analyzed for the first time. The unbound C_max_ in patients with ADRs (reversible visual changes; neurological abnormity or mental abnormity, such as nightly excitement, lack of alertness, and unconsciousness) were significantly higher than those without ADRs, while there was no similar result in C_t_, which indicated that C_u_ showed a more sensitive relationship with ADRs than C_t_.

The protein binding characteristics of VRC was also investigated in our study. The PPB was significantly higher in patients than all the *in vitro* samples, which was consistent with Florent et al. (Florent et al., 2014) reported in 2014, indicating that protein binding characteristics of VRC is different between real-life setting samples and *in vitro* spiking samples.

PPB was higher in sample with higher C_t_ or higher protein concentration. This positive relationship occurred both in patient plasma samples and in in-vitro samples, and was more obvious in AAG samples. Besides, the AAG showed the highest affinity with VRC compared with another two proteins, since there is little difference in the binding rate between 1 g/L AAG solution samples and 50 g/L ALB solution samples or 30 g/L GLB solution samples. This is not consistent with previous study (Vanstraelen et al.) that VRC was shown to bind preferably to ALB. It may be caused by the different device used for equilibrium dialysis. Therefore, AAG may play an important role in the protein binding characteristics of VRC when ALB is in an abnormally lower concentration.

Another notable result was that the binding rate of the protein mixture is about 50%-60%, although the protein binding rate of VRC with three different plasma proteins was almost 30%-50% respectively. Thus, the relationship between total PPB and PPB of individual protein is not a simple addition, but a compressive combination.

In this study, equilibrium dialysis (ED), the most common methods was used for investigating drug protein binding characteristics. ED is typically considered to be the gold standard (Zhang et al., 2012), using a semi-permeable membrane to separate unbound and bound drug, minimizing nonspecific binding of compounds to the device and avoiding the need for large plasma volumes (Zhang et al., 2012). We also have established a HPLC-MS/MS method for drug determination, and designed a series of experiments *in vitro* and *in vivo* to investigate the changing characteristics of C_u_ and PPB, in order to provide guidance for clinical medication. More cases should be taken into analysis in the future study.

## Conclusions

VRC plasma protein binding rate in patient was significantly higher (69.5 ± 6.2%) than that in in-vitro samples, influenced by C_t_, plasma protein concentration, and protein type. The α1-acid glycogen has the highest affinity with VRC. Relationship between total PPB of VRC with PPB of individual protein is not a simple addition, but a compressive combination. Unbound drug concentration of VRC shows relationships with C_t_, protein concentration, AST level, metabolism type of CYP2C19, co-administration of high PPB medicine. The relationship between unbound drug concentration and ADRs is more sensitive than that total plasma concentration do.

## Data Availability Statement

The datasets generated for this study are available on request to the corresponding authors.

## Ethics Statement

The studies involving human participants were reviewed and approved by the Ethical Committee of Jiangsu Province Hospital. Written informed consent to participate in this study was provided by the participants' legal guardian/next of kin.

## Author Contributions

Y-QW, X-YZ, LM, and X-HZ designed the clinical trial. Z-Q-YY, L-NS, Z-CY, and YQ performed the drug concentration analyses. QC and LY performed the genetic analyze. Z-Q-YY performed the statistical analyses. All authors were involved in drafting or reviewing the manuscript and approved the final version.

## Funding

This project was sponsored by the grants from the Priority Academic Program Development of Jiangsu Higher Education Institutions, National Natural Sciences Foundation of China (81673515, 81870436, 81503160), Natural Science Foundation of Jiangsu Province (BK20161591), Six Talent Peaks Project in Jiangsu Province (2014-YY-001), Jiangsu Provincial Medical Youth Talent (QNRC2016215), Suzhou science and education Youth Project (KJXW2016067), and Suzhou industrial technology innovation (SYSD2016046).

## Conflict of Interest

The authors declare that the research was conducted in the absence of any commercial or financial relationships that could be construed as a potential conflict of interest.
